# Individuals' willingness to pay for HIV vaccines in Iran: Contingent valuation method

**DOI:** 10.1002/hsr2.70016

**Published:** 2024-08-28

**Authors:** Sanaz Parvizi, Mohsen Mehrara, Ali Taiebnia

**Affiliations:** ^1^ Faculty of Economics University of Tehran Tehran Iran

**Keywords:** aids, contingent valuation design, HIV, vaccine, willingness‐to‐pay

## Abstract

**Background and Aims:**

This study aims to assess individuals' willingness to pay (WTP) for a human immunodeficiency virus (HIV) vaccine in Iran, focusing on key determinants influencing vaccine acceptance. Utilizing the contingent valuation method (CVM), we aim to provide insights into the economic and socio‐cultural factors shaping WTP in Iran.

**Methods:**

We conducted face‐to‐face surveys with 468 participants in Tehran province using purposive sampling. We analyzed demographic characteristics, perspectives on HIV/AIDS, and financial considerations alongside participants' WTP. Statistical analyses, including maximum likelihood estimation, identified factors influencing WTP. Mean WTP values were calculated to quantify the economic valuation of an HIV vaccine.

**Results:**

Higher education and monthly income significantly correlated with increased WTP, while gender, age, marital status, and health insurance showed no substantial impact. The mean WTP was 12,590.83 to 12,599.63 thousand Rials in different models, both showing statistically significant WTP values.

**Conclusion:**

Higher education and income levels are key determinants of WTP for an HIV vaccine in Iran. These findings provide valuable insights for policymakers to enhance vaccine accessibility and acceptance in Iran.

## 1 INTRODUCTION

The human immunodeficiency virus (HIV) is a formidable pathogen that has cast a long shadow over global public health since its discovery in the early 1980s.^1^ Belonging to a family of viruses known as retroviruses, HIV is characterized by its ability to reverse the usual flow of genetic information from DNA to RNA. Its unique properties and devastating consequences make it one of the most extensively studied and challenging viruses in the history of medicine.^2^

HIV exists in two main types: HIV‐1 and HIV‐2. Of these, HIV‐1 is the most prevalent and virulent worldwide. The virus attacks and weakens the human immune system, specifically targeting CD4 + T cells, a type of white blood cell critical to the immune response.^3^ Over time, untreated HIV infection can lead to acquired immunodeficiency syndrome (AIDS), a condition characterized by severe immune system dysfunction. This weakened immune system leaves individuals susceptible to opportunistic infections and certain types of cancer, ultimately resulting in a shortened lifespan.

HIV transmission primarily occurs through the exchange of certain bodily fluids, including blood, semen, vaginal fluids, rectal fluids, and breast milk. Common modes of transmission include unprotected sexual intercourse, sharing of contaminated needles among drug users, and from mother to child during childbirth or breastfeeding.^4^ HIV can also be transmitted through blood transfusions, although this risk has been dramatically reduced through rigorous screening of blood donations.

The consequences of HIV infection are not confined to the realm of public health but extend to the social, economic, and psychological dimensions of affected individuals and communities.^5^ HIV/AIDS remains one of the most significant global health challenges, affecting millions of people worldwide. In Iran, as in many other countries, the prevalence of HIV continues to be a matter of concern, necessitating comprehensive strategies for prevention and control.

Recent data indicate that the number of reported HIV cases in Iran has been on the rise. The main routes of HIV transmission in Iran are through intravenous drug use, accounting for a significant proportion of new infections, followed by sexual transmission. This shift underscores the evolving nature of the epidemic in the country and the need for targeted interventions.

Stigma and discrimination associated with HIV/AIDS remain significant barriers to effective prevention and treatment efforts in Iran. These socio‐cultural challenges hinder individuals from seeking testing and treatment services, further complicating public health efforts to control the epidemic.

Vaccination has proven to be a highly effective tool in combating infectious diseases, and the development of an HIV vaccine has long been a priority in the global health community. Understanding the willingness of individuals to pay for such a vaccine is essential for shaping future vaccination programs and policies.

The concept of “willingness to pay” (WTP) is a crucial economic indicator used to assess the monetary value individuals place on various goods and services.^6^ In the context of healthcare, WTP studies are instrumental in determining the perceived value of preventive measures, treatments, or interventions. These studies provide invaluable insights into individuals' preferences and priorities, aiding policymakers and healthcare professionals in resource allocation and decision‐making.^7^

This manuscript focuses on examining individuals' WTP for an HIV vaccine in Iran, for our goal Tehran province, the capital and most populous region of Iran, was selected as the study setting due to its unique characteristics. As the political, economic, and cultural heart of the country, Tehran offers a diverse population that mirrors the broader demographic trends of Iran. This diversity is essential for conducting a comprehensive analysis of the factors influencing WTP for an HIV vaccine. Moreover, Tehran's status as a central hub for healthcare services and policy implementation makes it a strategic location for this study. Insights gained from this region can inform national strategies and enhance the generalizability of the findings to other parts of the country.

To explore individuals' WTP for an HIV vaccine in Iran, we employ the Contingent Valuation Method (CVM). CVM is a widely accepted approach for estimating the economic value of nonmarket goods or services,^8^ such as healthcare interventions, by directly eliciting individuals' preferences and their associated monetary values.

In the following sections of this manuscript, we will discuss the methodology employed in our study, present the results of our survey and analysis, and conclude with a discussion of the implications of our findings for healthcare policy and practice in Iran. By examining the willingness of individuals to invest in HIV prevention through vaccination, we aim to contribute to the ongoing efforts to combat this global health challenge and improve public health outcomes in Iran.

## 2 METHODS

This research involves the use of the CVM to evaluate individuals' WTP for HIV vaccines within the Iranian population. CVM is a widely recognized and accepted approach for assessing the economic value that individuals place on goods or services, particularly when no market prices exist.^8^, ^9^ In the context of healthcare, it is a valuable tool for estimating the perceived value of health‐related interventions, such as vaccines.^7^, ^10^, ^11^, ^12^

The CVM was selected for this study because it is the most suitable approach for assessing individuals' WTP for HIV vaccines in Iran when no market prices exist. CVM's unique ability to capture hypothetical WTP in scenarios where such vaccines are not yet available is pivotal in estimating their perceived economic value to the Iranian population. This methodological choice allows us to comprehensively evaluate the economic worth of HIV vaccines, providing essential data for evidence‐based policymaking and resource allocation in public health.

### 2.1 Survey design

In this study, we employed a face‐to‐face data collection method, facilitated by a team of trained interviewers, to investigate individuals' WTP for HIV vaccines in the context of Iran. The data collection phase spanned 3 months, commencing in September and concluding in November 2023. Our target population consisted of Iranian citizens aged 18 years and above who were residing in Tehran province, chosen for its diverse population and representativeness.

To enhance the robustness of our methodology, the survey instrument underwent a meticulous design process and pre‐testing phase. Initially, a comprehensive literature review informed the creation of a draft questionnaire focusing on key factors influencing WTP for health interventions, particularly HIV vaccines. This draft was reviewed by an expert panel in public health, epidemiology, and health economics to ensure clarity, relevance, and cultural appropriateness. Subsequently, the questionnaire was pre‐tested with a sample of 30 participants from Tehran province, representative of the broader population. The pre‐test aimed to assess the clarity and comprehension of the questions, identify ambiguities, and refine the overall structure and flow of the survey. Feedback from this phase led to minor adjustments, ensuring that the final questionnaire was user‐friendly and effective in capturing the necessary data. This thorough approach to survey design and pre‐testing significantly strengthened the validity and reliability of our data collection instrument.

The final version of the questionnaire was administered to a larger sample of 468 participants in Tehran province. The questionnaire employed in this study was divided into three distinct sections, each playing a vital role in our investigation into individuals' WTP for HIV vaccines in the Iranian context. The first section was dedicated to gathering crucial information about respondents' knowledge and experiences related to the HIV virus as well as their previous encounters with vaccination. The second section of the questionnaire included inquiries about the demographic and socioeconomic status of the respondents. These questions were designed to provide a comprehensive profile of the study's participants, including details about their age, gender, education level, income, and other key factors that could influence their WTP for a vaccine. The final section of the questionnaire featured two key components. The first subsection introduced participants to a contingent market scenario, followed by a question inquiring about their WTP for an HIV vaccine. The second component of this section focused on understanding the reasons behind individuals' unwillingness to pay for a vaccine.^12^, ^13^, ^14^, ^15^ Participants were encouraged to articulate various factors, whether economic, social, or personal, that influenced their decision to abstain from contributing to the hypothetical vaccine. Ethical considerations were central to our data collection process, with informed consent obtained from all participants, and assurances of confidentiality and the right to withdraw were provided. Rigorous measures were implemented to ensure data quality and control, and data security protocols were followed to protect the anonymity of participants.

To ascertain the necessary sample size for our study, we applied a well‐established formula,

(1) $$n=\frac{\;z^{2}\text{pq}}{e^{2}}=\frac{1.96^{2}(0.5\times 0.5)}{0.05^{2}}=385,$$

where ‘*n*’ represents the required sample size, ‘*Z*’ is the standard normal value corresponding to the desired confidence level (1.96 for a 95% confidence interval), ‘*p*’ denotes the probability of success (0.5), ‘*q*’ signifies the probability of failure (1 − p, also 0.5 in our case), and ‘*e*’ stands for the margin of error (5%).^13^, ^16^, ^17^, ^18^ Employing these parameters, our initial calculation yielded a minimum required sample size of 385. However, to enhance the robustness of our study, we opted to collect 468 questionnaires during the data collection phase, surpassing the calculated minimum requirement. This decision was made to enhance the reliability and generalizability of our study's results. The larger sample size not only provides a more extensive data set but also offers increased statistical power.

### 2.2 Selecting variables

To select variables, our study commenced with a thorough review of existing literature on HIV vaccine acceptance and WTP across various global and regional contexts.^1^, ^3^, ^5^ This literature review not only highlighted key factors influencing vaccine acceptance for HIV but also drew upon studies examining WTP for vaccines against other diseases.^13^, ^14^, ^15^, ^17^, ^18^ This comprehensive approach ensured that we considered a broad range of factors influencing vaccine adoption and financial considerations pertinent to Iran's healthcare landscape. In addition to the literature review, we engaged with experts in health economics, public health policy, and epidemiology who are familiar with Iran's healthcare landscape. Their expertise was instrumental in identifying critical variables essential for assessing WTP for HIV vaccines in a socio‐cultural and economic context specific to Iran. Before formal data collection, we conducted a pilot study to validate the selected variables within the Iranian population. This preliminary phase ensured that the variables were comprehensible and relevant to potential study participants, thereby enhancing the robustness of our data collection strategy.

The culmination of our literature review, expert consultations, and pilot testing informed the selection of the following variables for our study:

**Gender:** Refers to the categorization of individuals as male or female.

**Age:** Represents the chronological age of individuals.

**Marital status**: Indicates whether individuals are single, married, divorced, or widowed.

**Health insurance**: Refers to the presence or absence of insurance coverage for healthcare services.

**Risk of contracting the HIV**: Assesses individuals' perception of their likelihood of acquiring HIV.

**Are there any AIDS patients:** Refers to the presence of AIDS Patients with individuals who answer survey.

**Have underlying health conditions**: Refers to pre‐existing medical conditions individuals may have.

**Experienced side effects from other vaccines**: Refers to any adverse reactions individuals may have had from previous vaccinations.

**Substantial financial expense:** Includes the financial burden associated with vaccination, such as vaccine cost and affordability relative to individuals' income levels.

**Information HIV**: Refers to knowledge about HIV virus such as transmission and prevention methods.

**Hear HIV**: Refers to hear about HIV vaccine.

It is important to note that all statistical analyses were executed using Stata software, with a specific focus on version 17. The threshold for statistical significance was established at *p* < 0.05. Categorical data representing individual characteristics were conveyed in terms of frequencies and percentages (*n*, %).

### 2.3 Approval for ethical conduct and participant assent

The authors of this study have meticulously followed established ethical guidelines, which encompass compliance with the 2013 revised Declaration of Helsinki, to ensure the rigorous collection and accurate reporting of data. Additionally, it is crucial to emphasize that the participation of individuals in our surveys was entirely voluntary, and their informed consent was diligently obtained. The study upheld the principles of voluntary participation and informed consent as central tenets, prioritizing the rights and autonomy of our valued participants.

### 2.4 Econometric analysis and determining WTP

In our study, we employed a double‐bounded dichotomous choice format for contingent valuation questions to elicit individuals' WTP for an HIV vaccine. Survey participants were initially presented with a randomly assigned initial price bid (bidi) and asked whether they would purchase the vaccine at that price. If they responded affirmatively, they were subsequently queried about their willingness to buy the vaccine at a higher price bid (bidH). Conversely, for those who indicated unwillingness to purchase the vaccine at the initial price, a lower price bid (bid L) was offered. The initial bid used in these questions was derived from a pre‐survey involving 70 individuals who expressed their WTP for a vaccine in an open‐ended format. We applied an iterative approach to estimate the bid vector, with the goal of minimizing the mean square error of the bid design. This process was guided by the assumption of a log‐normal distribution with a limit of 5 bid values (bidi).^8^, ^13^, ^14^, ^19^ From these bid values, we determined the upper (bid H) and lower limits (bid L) for each by adding or subtracting half of its value, respectively,^13^, ^14^ (see Table 1 for details).

**Table 1:** payment vecrtor (thousand Rials).

Initial bid	Bid low	Bid high
1430	715	2145
3300	1650	4950
6450	3225	9675
12,600	6300	18,900
29,000	14,500	43,500

*Note*: Rials 521,500 = US$1 at the time of the study.

The responses to the bid value scenarios resulted in four possible response patterns:

(1) Bid H ≤ WTP (yes, yes)
(2) Bidi ≤ WTP ≤ Bid H (yes, no)
(3) Bid L ≤ WTP ≤ Bidi (no, yes)
(4) WTP ≤ Bid L (no, no)

The econometric estimation is based on the assumption that the WTP can be represented as follows:

(2) $${\mathrm{WTP}}_{i}\left(z_{i},u_{i}\right)=z{’}_{i}b+u_{i}\;and\;u_{i}\approx N\left(0,\delta^{2}\right)$$

where *z_i_* represents a vector of explanatory variables, *b* represents a vector of parameters, and *u_i_* represents an error term approximately following a normal distribution with a mean of 0 and variance *δ* ^2^.

The parameters of the model were estimated by maximizing the following function:

(3) $$\sum_{i=1}^{N}[d_{i}^{\text{yn}}\ln\left(\varphi \left(z_{i}^{\prime }\frac{\beta }{\sigma }-\frac{{\mathrm{Bid}}_{H}}{\sigma }\right)-\varphi \left(z_{i}^{\prime }\frac{\beta }{\sigma }-\frac{{\mathrm{Bid}}_{i}}{\sigma }\right)\right)+d_{i}^{\text{yy}}\ln(\varphi \left(z_{i}^{\prime }\frac{\beta }{\sigma }-\frac{{\mathrm{Bid}}_{H}}{\sigma }\right))+d_{i}^{\text{ny}}\ln\left(\varphi \left(\;z_{i}^{\prime }\frac{\beta }{\sigma }-\frac{{\mathrm{Bid}}_{i}}{\sigma }\right)-\varphi \left(\;z_{i}^{\prime }\frac{\beta }{\sigma }-\frac{{\mathrm{Bid}}_{L}}{\sigma }\right)\right)+d_{i}^{\text{nn}}\;\ln(\;1-\varphi \left(z_{i}^{\prime }\frac{\beta }{\sigma }-\frac{{\mathrm{Bid}}_{L}}{\sigma }\right))]$$

In our analysis, we introduced four indicator variables, denoted as $d_{i}^{\text{yn}},d_{i}^{\text{yy}},d_{i}^{\text{ny}}$, and $d_{i}^{\text{nn}}$, which assume binary values of one or zero. These indicators are employed to categorize respondents into specific cases, with each individual contributing to the likelihood function's logarithm in only one of the four possible parts. This approach allows us to effectively model the diverse responses from the participants. Subsequently, we estimated the parameters *β* and *σ* using the maximum likelihood method, following the approach introduced by Lopez–Feldman.^8^, ^12^, ^17^, ^20^, ^21^, ^22^

In the following step, we calculated the mean WTP, denoted as $E\left(\mathrm{WTP}|Z\right)=\;\bar{Z\prime }\;\hat{\beta }$. Here, $\bar{Z\prime }\;$signifies a vector containing the weighted average demographic characteristics of the participants. We determined E(WTP|Z)by taking the inner product of $\bar{Z\prime }$ and the maximum likelihood parameter estimate, symbolized as $\hat{\beta }$, which was obtained in the initial stage.

## 3 RESULTS

### 3.1 The respondents' characteristics and their awareness of HIV virus

#### 3.1.1 Demographic overview

The gender distribution in our study reveals a predominant representation of females, constituting 68%, while males account for 32%. Within the age groups, the 30–39 category is the most prevalent at 39%, followed by 40–49 (19%), 50–59 (15%), and those aged 60 or above (11%). In terms of education, 40% of participants have attained a Bachelor's degree, showcasing educational diversity. Monthly income is varied, with 70% reporting earnings exceeding 50,000,000 IRR. Marital status reflects a majority of participants being married (70%), with 25% single and 5% divorced or widowed. Health insurance coverage is reported by a substantial 73% of participants.

#### 3.1.2 Perspectives on HIV/AIDS

The data indicates that 81% of participants believe there is no risk of contracting HIV. Additionally, the majority (88%) reports no individuals with AIDS in their vicinity, suggesting a prevalent perception of low local prevalence. Notably, 36% of participants report underlying health conditions, highlighting a significant consideration in health‐related decision‐making. Encouragingly, 89% have not experienced side effects from other vaccines that would deter them from getting the AIDS vaccine.

Financial considerations emerge as a notable factor, with 44% of participants expressing the belief that vaccination is hindered by substantial financial expense. This underscores the importance of addressing economic barriers to enhance vaccine accessibility. On the knowledge front, a substantial 71% of participants feel sufficiently informed about HIV, and 64% possess heard about the HIV vaccine (Table 2).

**Table 2:** Demographic and social aspects related to individuals.

	*N*	%		*N*	%
Gender			Is there risk of contracting the HIV virus?		
Male	152	32	Yes	89	19
Female	316	68	No	379	81
Age			Have there been any individuals with AIDS in your vicinity, either currently or in the past?		
18–29	77	16	Yes	54	12
30–39	182	39	No	414	88
			Do you have any underlying health conditions such as high blood pressure, cancer, or diabetes?		
40–49	90	19	Yes	170	36
50–59	70	15	No	298	64
≥60	49	11	Have you experienced mild or severe side effects from other vaccines that would deter you from getting the AIDS vaccine?		
Education			Yes	50	11
≤Diploma	96	20	No	418	89
Diploma	115	25	Is the vaccination hindered by the substantial financial expense?		
Bachelor	186	40	Yes	206	44
Master	39	8	No	262	56
PHD	32	7	Are you sufficiently informed about the HIV virus?		
Income monthly			Yes	330	71
≤50,000,000 IRR	36	7	No	138	29
50,000,000–100,000,000 IRR	130	28	Do you have heard about the HIV vaccine?		
100,000,000–200,000,000 IRR	163	35	Yes	298	64
≥200,000,000 IRR	139	30	No	170	36
Marital status					
Married	325	70			
Single	118	25			
Divorced/widow	25	5			
Health insurance					
Yes	343	73			
No	125	27			

**Figure 1:**
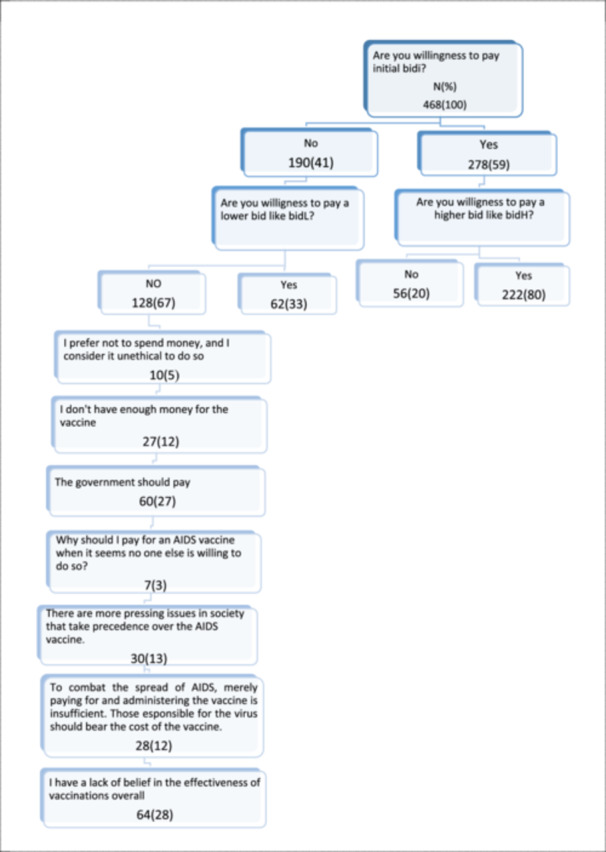
Summary of statistics for responses to the double‐bounded dichotomous choice questions.

In the survey involving 468 participants assessing their WTP the initial bid for an AIDS vaccine, 59% agreed to pay. Notably, Among those who said “yes” to the initial bid, 80% were willing to accept a higher bid (bidH), while 20% rejected the proposition. Conversely, for a lower bid (bidL), 33% agreed to contribute, while 67% rejected the offer. Among those who rejected, reasons varied, with 5% expressing ethical concerns, 12% citing financial constraints, and 27% suggesting that the government should bear the cost. A smaller fraction (3%) questioned the necessity of individual payment. Additionally, 13% prioritized other societal issues, and 28% expressed a general rejection of the effectiveness of vaccination (Figure 1).

### 3.2 Influencing factors on the WTP for an HIV vaccine

Table 3 displays the outcomes of the maximum likelihood estimation (Equation 3) assessing the factors influencing the WTP for an HIV vaccine. The results show that gender exhibited no statistically significant associations with WTP, indicating a neutral impact on individuals' decisions. The non‐significance of gender in determining WTP could be attributed to a shared understanding and prioritization of health across both genders. In our sample, both males and females might have similar concerns about HIV and view the vaccine as equally important, leading to no significant difference in WTP. In addition, The age of participants did not significantly impact WTP, possibly because health awareness and the perceived importance of the vaccine are consistent across different age groups in our sample. This suggests a uniform perception of the vaccine's value, regardless of age. On the contrary, educational background emerged as a crucial factor (*p* < 0.001), indicating that individuals with higher education levels are more WTP for HIV vaccine compared to their less‐educated counterparts. Marital status did not influence WTP (*p* = 0.75) significantly, potentially because the decision to pay for the vaccine is seen as an individual health responsibility, rather than one influenced by marital or household dynamics. Importantly, the results highlight a statistically significant relationship between monthly income and WTP (*p* = 0.03), suggesting that higher income levels positively influence the WTP for an AIDS vaccine. However, the presence of health insurance did not significantly affect WTP (*p* = 0.15) due to a general belief that the vaccine should be a public health provision, making the presence or absence of insurance less relevant in the decision‐making process for paying out‐of‐pocket.

**Table 3:** WTP estimation: maximum likelihood estimation of the parameters *β* and *σ*.

Attributes	Coefficients	Standard error	*z*	*p*‐Value	95% Confidence interval
Gender	−1050.963	2122.196	−0.50	0.62	−5210.391, 3108.465
Age	685.2738	985.7983	0.70	0.49	−1246.855, 2617.403
Education	4167.264	984.7629	4.23	<0.001	2237.164, 6097.364
Marital status	649.4757	2081.388	0.31	0.75	−3429.97, 4728.922
Income monthly	2428.713	1125.206	2.16	0.03	223.3498, 4634.076
Health insurance	3208.946	2247.303	1.43	0.15	−1195.686, 7613.578
Risk of contracting the HIV	2338.889	2660.207	0.88	0.38	−2875.021 7552.798
Are there any AIDS patients	6113.586	3306.823	1.85	0.06	−367.668, 12594.84
Have underlying health conditions	−1948.653	2219.783	−0.88	0.38	−6299.348, 2402.042
Experienced side effects from other vaccines	−3568.701	3137.843	−1.14	0.25	−9718.759, 2581.358
Substantial financial expense	−3169.422	1934.422	−1.64	0.10	−6960.819, 621.9758
Information HIV	3337.897	2217.843	1.51	0.13	−1008.996, 7684.79
Hear HIV	−279.0377	2064.267	−0.14	0.89	−4324.927, 3766.852
_cons	−10,507.51	5466.794	−1.92	0.05	−21,222.23, 207.2072
Sigma					
_cons	17,053.34	1208.105	14.12	< 0.001	14685.5, 19421.18

*Note*: for *p*‐values less than 0.001, report “*p* < 0.001”; for *p*‐values between 0.001 and 0.01, report the value to the nearest thousandth; for *p*‐values greater than or equal to 0.01, report the value to the nearest hundredth. Log likelihood = −595.83642, Wald *χ* ^2^ = 50.14, Prob > *χ* ^2^ = 0.0000, Number of obs = 468.

Abbreviations: HIV, human immunodeficiency virus; WIP, willingness to pay.

Turning our attention to health‐related attributes, the results show that the perceived risk of contracting HIV did not significantly affect WTP (*p* = 0.38), which could be due to a low perceived personal risk or a high level of confidence in existing preventive measures among the participants. Similarly, knowing someone with AIDS did not significantly change WTP (*p* = 0.06), suggesting that personal connections to HIV/AIDS patients do not strongly influence the economic decision to invest in vaccination. This may reflect a community‐wide perception of the vaccine's benefits rather than an individual risk‐based decision.

Similarly, underlying health conditions and prior experiences with side effects from other vaccines did not attain statistical significance (*p* = 0.38 and *p* = 0.25, respectively). The presence of underlying health conditions did not impact WTP significantly, which might be because participants prioritize immediate health concerns over preventive measures, or they perceive the vaccine as equally necessary regardless of existing health conditions. In addition, previous experiences with side effects from other vaccines did not significantly deter WTP, indicating that such experiences do not heavily weigh against the perceived benefits of the HIV vaccine.

Considering economic and knowledge‐related attributes, the results suggest that financial concerns, while important, did not significantly differentiate WTP, possibly because participants may expect public health programs to subsidize the cost of the vaccine or because the perceived benefit outweighs the financial burden. Conversely, knowledge about HIV and awareness of the vaccine did not significantly influence WTP (*p* = 0.13 and *p* = 0.89, respectively), because awareness alone does not translate into WTP without accompanying perceived benefits and necessity.

### 3.3 Mean WTP for HIV vaccine

Table 4 presents the estimated mean WTP for both the basic and expanded models. The basic model assumes the absence of control variables, while the expanded model incorporates these variables to establish the best‐fit model. The results highlight the mean WTP for the HIV vaccine in both models.

**Table 4:** Mean willingness to pay for HIV vaccine.

WTP	Mean WTP	Standard error	*z*	*p*‐Value	95% Confidence interval
Basic model	12,590.83	1056.776	11.91	<0.001	10,519.59, 14,662.08
Expanded model	12,599.63	982.8287	12.82	<0.001	10,673.32, 14,525.93

*Note*: For *p* values less than 0.001, report “*p* < 0.001”; for *p* values between 0.001 and 0.01, report the value to the nearest thousandth; for *p* values greater than or equal to 0.01, report the value to the nearest hundredth. *z *= normal test estimated, assuming 95% confidence.

Abbreviations: HIV, human immunodeficiency virus; WIP, willingness to pay.

The mean WTP for the HIV vaccine was 12590.83 (Thousand Rials) in the basic model, with a 95% CI ranging from 10,519.59 to 14,662.08. In the expanded model, the mean WTP slightly increased to 12,599.63 (Thousand Rials), and the 95% confidence interval ranged from 10,673.32 to 14,525.93. Both models exhibited statistically significant estimated WTP values.

These findings emphasize the influence of control variables in refining our understanding of individuals' WTP for an HIV vaccine. The robust statistical significance in both models provides a comprehensive foundation for policymakers and healthcare strategists to design targeted interventions and optimize the effectiveness of HIV vaccination programs.

## 4 DISCUSSIONS

Our investigation into the WTP for an HIV vaccine among 468 participants in Iran, utilizing the CVM, unveils a complex interplay of factors influencing vaccine acceptance.

The analysis revealed that neither gender nor age exhibited statistically significant correlations with individuals' WTP for an HIV vaccine. However, in contrast, the statistically significant association with educational level underscores its pivotal role as a significant determinant of individuals' WTP for the HIV vaccine. Individuals with higher educational attainment demonstrated a greater inclination to contribute financially to the vaccine, emphasizing the influential role of education in shaping perceptions and attitudes. This finding highlights the importance of considering educational backgrounds in understanding and promoting vaccine acceptance.

Interestingly, monthly income exhibited a notable positive correlation with participants' WTP for an HIV vaccine. The findings suggest that individuals with higher monthly incomes demonstrated a greater propensity for WTP. Specifically, participants earning within the bracket of (more than 200,000,000 IRR) displayed a higher WTP for the HIV vaccine compared to the reference group with a monthly income of (less than 50,000,000 IRR). This positive correlation emphasizes the influence of income levels on vaccine acceptance and suggests that economic considerations play a pivotal role in shaping individuals' decisions regarding HIV vaccination.

In our investigation, we discovered that neither marital status nor health insurance significantly influenced individuals' WTP for an HIV vaccine, highlighting the independent nature of vaccination decisions. Despite exploring health‐related aspects such as perceived risk, the presence of AIDS patients, underlying health conditions, and experiences with side effects, no substantial impact on WTP was observed. Unexpectedly, knowledge about HIV and awareness of the vaccine did not significantly influence individuals' WTP.

Examining the acceptance rate, 59% of participants expressed a WTP the initial bid for the HIV vaccine. Within this group, 80% were open to accepting a higher bid, while 20% rejected the proposition. Conversely, for a lower bid, 33% agreed to contribute, while 67% rejected the offer. Reasons for rejection varied, with ethical concerns, financial constraints, and the belief in government responsibility being prominent factors.

Turning to the mean WTP values, the basic model yielded an estimated mean WTP of 12,590.83 Thousand Rials, with a 95% CI ranging from 10,519.59 to 14,662.08. The expanded model, incorporating demographic and socioeconomic control variables, slightly adjusted the mean WTP to 12,599.63 Thousand Rials, with a confidence interval between 10,673.32 and 14,525.93. These statistically significant mean WTP values underscore the perceived economic value attributed to an HIV vaccine by the Iranian population.

In the absence of a dedicated body of literature on the WTP for HIV vaccines, our study contributes to the broader understanding of vaccine acceptance by drawing parallels with research conducted in related fields, such as influenza, COVID‐19, HPV, and… vaccines. While the direct comparison may not fully capture the unique dynamics of HIV vaccine acceptance, it provides a valuable framework for exploring commonalities and differences in individuals' attitudes towards preventive measures.

Our study's method for estimating WTP for an HIV vaccine closely mirrors the approaches employed in related research, as seen in studies conducted by García and Arcadio,^14^ Soofi et al.,^17^ and Cerda and García.^13^ These investigations, like ours, utilized the CVM in its double‐bounded dichotomous choice format. This alignment in methodology strengthens the reliability and comparability of our results across studies, contributing to a more comprehensive understanding of economic valuation in the context of vaccine acceptance research.

The positive correlation between monthly income and WTP uncovered in our study signifies that individuals with higher monthly income levels are more inclined to express a WTP for an HIV vaccine. This aligns cohesively with the outcomes observed in multiple studies,^13^, ^14^, ^17^ alluding to a consistent pattern where higher income is associated with a greater WTP in various vaccines. The implication of this correlation is profound, as it underscores the pivotal role of economic considerations in shaping individuals' decisions regarding vaccination.

Our study reveals that marital status, age, and gender do not exert a significant influence on WTP, emphasizing the autonomy individuals maintain in vaccination decisions, irrespective of their relationship status. This finding is consistent with the suggestions in References,^14^, ^17^ although those studies were focused on COVID‐19. And also, Our results align with previous research highlighting the crucial role of educational background in shaping individuals' WTP for vaccinations.^13^, ^17^, ^18^ This suggests that individuals with higher educational attainment are more likely to pay in healthcare measures, possibly due to an increased awareness and understanding of the importance of preventive health practices.

### 4.1 Limitation

An important limitation of our study is that we did not investigate factors such as ethical concerns and broader societal issues that could potentially influence individuals' WTP for an HIV vaccine in Iran. Understanding these dimensions could provide a more comprehensive understanding of vaccine acceptance dynamics and enhance the effectiveness of public health interventions and policies. Factors such as trust in healthcare systems, community perceptions, and ethical perspectives on vaccine distribution are likely to influence vaccine acceptance and should be investigated more comprehensively in future research. Additionally, long‐term studies could assess how these factors evolve over time and in different demographic groups, contributing to a deeper understanding of vaccine uptake. By addressing these dimensions, future studies can provide a more holistic understanding of vaccine acceptance dynamics, thereby informing more effective public health interventions and policies.

## 5 CONCLUSIONS

Our study provides valuable insights into the factors influencing individuals' WTP for an HIV vaccine in Iran, highlighting the critical role of socioeconomic status in vaccine acceptance. To enhance vaccine uptake, policymakers should consider implementing targeted subsidies or financial support programs for lower‐income individuals to make the vaccine more affordable. Additionally, educational and awareness campaigns focusing on the benefits of HIV vaccination are essential, particularly for less‐educated populations. Community engagement and tailored messaging can address socio‐cultural barriers, while expanding health insurance coverage to include HIV vaccines can reduce out‐of‐pocket expenses, further increasing accessibility. By addressing the financial and educational barriers identified in this study, policymakers can develop effective strategies to improve HIV vaccine acceptance and uptake, ultimately contributing to better public health outcomes and the control of HIV spread in Iran.

## AUTHOR CONTRIBUTIONS

**Sanaz Parvizi**: Conceptualization; formal analysis; investigation; validation; software; methodology; visualization; writing—original draft; writing—review and editing; resources; data curation. **Mohsen Mehrara**: Conceptualization; formal analysis; investigation; methodology; visualization; validation. **Ali Taiebnia**: Conceptualization; investigation; methodology; validation; visualization; formal analysis.

## CONFLICT OF INTEREST STATEMENT

The authors declare no conflicts of interest.

## TRANSPARENCY STATEMENT

The lead author Sanaz Parvizi affirms that this manuscript is an honest, accurate, and transparent account of the study being reported; that no important aspects of the study have been omitted; and that any discrepancies from the study as planned (and, if relevant, registered) have been explained.

## Data Availability

The data that support the findings of this study are available from the corresponding author upon reasonable request.
